# Annotations

**Published:** 1888-02-04

**Authors:** 


### ANNOTATIONS.

**A Birching Machine.**

THE steamship was a mighty achievement, the railway and the electric telegraph were, if possible, more wonderful still. But the triumphs of modern science seem destined to know no end. The latest application of the electric current is one which could never have occurred to anybody but a man of genius. France is eminently the country of ideas, and sometimes of new ones. A brilliant Frenchman has invented an *electric birch*, which is said to be so sweetly and reasonably effective that no school boy who has once had experience of its skill will consent to be birched in any other way. This most just and gentlemanly birch does its work with such calm and dignified precision that no mark is ever left upon the most susceptible integument, nor does the fortunate birched one find the least difficulty in assuming any desired posture the moment the birching is done. So far as we know the instrument has not yet been tried in this country, nor do we imagine it is the least likely ever to become an established institution among us. A French boy may submit to the ignominy of taking a birching from a mechanical arrangement propelled by electricity, but if it were attempted to reduce an English schoolboy to such a base and degrading level, we should expect him to turn round upon the machine and smash it to atoms. It may be satisfactory to know that the new birch cannot warm up into a fine rage and give twice as severe a flogging as was originally intended; but most boys would prefer a birching of the worst possible severity from a man and a head master to the mildest application of the rod from a birching machine. No doubt machinery may do much for us—carry our messages, play our pianos, milk our cows, and make our cheeses; but we must draw a line somewhere, and we think it should be drawn at the birching machine. If the French inventor really wants to do a good service to school-boys, let him invent a machine which will carry its fortunate posssessor across the *pons asinorum* and through other preliminary difficulties of a like kind. We can promise him, in that case, unlimited patronage in every order of school in England, from Eton downwards.

**Hindu Medicine.**

WHY do not some of our medical men and medical women, who purpose practising in India, devote some study to the ancient Hindu medical literature? It would not be wasted time, if only for the knowledge they would gain of the tongue their patients will mostly speak, and in other respects it would be exceedingly valuable. The *Ayur-Veda* is by no means a despicable piece of literature, and that part of it which treats of medicine is well worth knowing. The books of Charaka and Susruta, though they were compiled not later than B.C. 300, are by no means the mere agglomeration of superstition which most of us rashly assume all ancient medicine to have been. On the contrary, they clearly prove that the high rank physicians held in the social system of Hindostan was fully justified by their practical knowledge of their art; and it must be remembered that India was one of the most cultured and enlightened nations of the ancient world till the Mohammedan conquest flooded it with barbarous Arab tribes and their descendants. The operations which the surgeons of old times successfully performed were such as demanded considerable skill, and a more thorough knowledge of anatomy than we are usually ready to credit them with, and mere fragments of their knowledge of chemistry have formed the foundation of the science of the Western world. The Hindus, as Dr. Wise shows in his "Review of the History of Medicine," knew the properties of a long list of medical plants and natural salts; were acquainted with the preparations of the alkalies, and of sulphuric, nitric, and muriatic acids; and by bringing them into contact, observed the complete alterations produced in their physical properties and chemical actions. This knowledge ultimately led them astray by inducing them to believe that all forms of matter are mutually convertible into each other, and so developed the theory of the transmutation of all metals into gold. This and kindred superstitions are found in the pages of the *Ayur-Veda*, and it is possible that the more materialistic of modern students would resent the combination of physic and metaphysic in Charaka's lectures to his students. But in spite of this, one cannot but admire the accurate diagnosis and classification of diseases shown by Charaka, and the reasonable treatment he recommends, while Susruta's anatomical descriptions and advice in matters of surgery may silence those who are too ready to disparage the wisdom of the ancients. Even in other matters the modern student may do worse than take the Hindu sage's advice not to degrade his profession by taking small fees, though it seems he means that the doctor should not be content with gold only, without "respect, gratitude, and love." But the practical value of these works lies in this, that they contain the gathered experience of centuries regarding the treatment of diseases under climatic conditions which are unfamiliar to the European doctor when first he goes to Hindostan. Dealing with the Asiatic development of ailments, whose English type alone he knows, or with some which he has never seen before, he might be saved from error by knowing the wisdom of the old sages. And again, he would find it easier to win the regard of his patients if they knew that he did not despise, but was familiar with, books for which they have a traditional reverence, and this in turn would make our Hindu fellow-subjects more ready to appreciate and profit by the increasing discoveries of Western medicine.

**Cottage Hospital Rules.**

RULES are excellent things, but they cannot be so constructed as to dispense altogether with reason in those who apply them. This general proposition has seldom received more effective, and at the same time more unsatisfactory, illustration than it did a short time ago at Oswestry. Mrs. Stringer, of Gate Street, Oswestry, left her little son, aged three years, at home in charge of his sister on Saturday afternoon, whilst she went out for a time. By some means the little fellow's clothing caught fire, and in his terror he ran out, all blazing, into the yard. A neighbour named Jones promptly extinguished the flames, and the child, in agonies of pain and terror, was carried to the Cottage Hospital; the most suitable place in the town one would naturally have thought. But the Oswestry Hospital has a code of regulations which evidently bears a close relationship to those "laws of the Medes and Persians" whose distinguishing characteristic was that they "altered not." According to the unalterable statutes in force at the Oswestry Cottage Hospital no child of three years is entitled to treatment at that institution. The poor infant, therefore, who had so recently been on fire, was refused admittance. Who could have been so unmotherly as to do such a thing? Was it the matron, or the hall-porter, or the chairman of committee? The rule which provides that young children are not to be received at the hospital may be quite necessary and quite wise. There may be no accommodation, and no nursing or other facilities for patients of such tender years. But there are cases where reason and humanity may properly prevail over rules, and where a law is more honoured in the breach than in the observance; and the burnt child of three, whose mother could not be found, was surely such a case. If the matron had consulted her motherly instinct she would have received the child, soothed his terror, and dressed his wounds. Then she might have sent for the mother, and explained to her the rule which prevented the boy remaining longer in the hospital. Thus, instead of a scandal, there would have been an act of kindness credited to the institution, and the only possible loss to it would have been a little lint and Carron oil. By all means let hospitals have rules, and let officials observe them. But it should never be forgotten that the rule of doing as we would be done by is a high statute which is permanent, universal, and always to be obeyed.

